# Leisure-Time Physical Activity and Academic Performance: Cross-Lagged Associations from Adolescence to Young Adulthood

**DOI:** 10.1038/srep39215

**Published:** 2016-12-15

**Authors:** Sari Aaltonen, Antti Latvala, Richard J. Rose, Urho M. Kujala, Jaakko Kaprio, Karri Silventoinen

**Affiliations:** 1 Department of Public Health, University of Helsinki, P.O. Box 20 (Tukholmankatu 8 B), FIN-00014 University of Helsinki, Finland; 2 Department of Social Research, University of Helsinki, P.O. Box 54, FIN-00014 University of Helsinki, Finland; 3 Department of Psychological and Brain Sciences, Indiana University, 1101 East 10th St., Bloomington, IN 47405, USA; 4 Department of Health Sciences, University of Jyvaskyla, P.O. Box 35, FIN-40014 University of Jyvaskyla, Finland; 5 Institute for Molecular Medicine (FIMM), University of Helsinki, P.O. Box 20 (Tukholmankatu 8), FIN-00014 University of Helsinki, Finland

## Abstract

Physical activity and academic performance are positively associated, but the direction of the association is poorly understood. This longitudinal study examined the direction and magnitude of the associations between leisure-time physical activity and academic performance throughout adolescence and young adulthood. The participants were Finnish twins (from 2,859 to 4,190 individuals/study wave) and their families. In a cross-lagged path model, higher academic performance at ages 12, 14 and 17 predicted higher leisure-time physical activity at subsequent time-points (standardized path coefficient at age 14: 0.07 (p < 0.001), age 17: 0.12 (p < 0.001) and age 24: 0.06 (p < 0.05)), whereas physical activity did not predict future academic performance. A cross-lagged model of co-twin differences suggested that academic performance and subsequent physical activity were not associated due to the environmental factors shared by co-twins. Our findings suggest that better academic performance in adolescence modestly predicts more frequent leisure-time physical activity in late adolescence and young adulthood.

There is evidence that consistent physical activity plays a crucial role not only in physical fitness, but also in psychological health^1^,^2^. It has been suggested that physical activity could also exert a positive effect on cognitive function and learning^3^,^4^,^5^. This putative association is based on evidence that physical activity^6^,^7^ as well as physical fitness^8^ are related to different types of changes in the brain relevant for cognitive function and learning. This has led to an increasing interest in the associations between physical activity behaviour and academic performance.

Several review articles have evaluated the association between physical activity and academic performance. However, no pooled effect sizes have been estimated in many of these reviews. Rasberry *et al*.^9^ reviewed some 50 cross-sectional and longitudinal studies, some with follow-ups of several years. They focused on the association between school-based physical activity and several indicators of school performance in children from 5 to 18 years of age; they concluded that the association between physical activity and academic performance was either positive or that no relationship could be demonstrated. Due to the small number and heterogeneity of the included studies, the magnitudes of effect sizes were not reported. Singh *et al*.^10^ reviewed the longitudinal studies concentrating on temporal associations between physical activity and academic achievement in young people also under the age of 18 years. The focus was on physical activity organized during school days, with most studies originating from North America. There was extensive variation of the intervention content between the studies with only two studies being evaluated of high methodological quality. A positive relationship between physical activity and academic performance was also found in this review, which the authors considered to be supportive of a causal effect. More recently, Mura *et al*. in 2015^5^ reviewed the effectiveness of school-based physical activity interventions on academic achievement. Despite different duration of interventions (range from 2 weeks to several years), physical activity carried out in school settings was found to have either a positive association (10 out 16 studies) or no association with academic performance (6 out of 16 studies).

The causality of these relationship can be studied best with randomized controlled trials and interventions, but they have been much rarely conducted in the field of physical activity and academic performance. Lees *et al*.^11^ concentrated on causal effects among children and adolescents under 19 years of age by summarizing eight randomized controlled trials using aerobic physical activity as an independent variable. The authors concluded that the results of these trials provided evidence that aerobic physical activity had a positive causal role in both cognitive functioning and academic achievement. However, the relationship was weak, because no statistically significant improvement had been detected in any of the individual studies included in the review. The validity of the included studies was assessed, but again, the opportunity to summarize the magnitudes of effect sizes was restricted. The authors speculated that some of the studies included in the review were underpowered and/or had too brief of duration of follow-up to be sufficiently reliable.

The findings emerging from these reviews based on dozens of studies suggest that the association between physical activity and academic performance is mainly positive, but there are other studies reporting no association or even a negative association. In particular, more recent studies using objectively measured physical activity have tended either to find no association^12^,^13^ or even a negative association^13^,^14^ between physical activity and academic performance. Some caution is necessary here since many factors, such as adequate statistical power and the type and duration of interventions, may contribute to the lack of association.

When reviewing the findings of the studies that examine the association between physical activity and academic performance, it is important to bear in mind that childhood intelligence and cognitive ability have shown to predict subsequent health^15^, physical activity^16^ and physical fitness^17^. For example in a British longitudinal study, higher mental ability at age of 10 years predicted a significantly higher level of physical activity in adulthood^16^. A more recent longitudinal New Zealand study showed that children with better cognitive ability achieved a higher level of physical fitness in adulthood^17^. Thus, the reported associations between physical activity and academic performance may partly reflect the associations between cognitive ability and physical activity.

No previous study has comprehensively investigated the mutual longitudinal associations between physical activity and academic performance to evaluate potential mechanisms underpinning the association. Despite suggestions of causality between physical activity and improved academic performance^11^, the direction of the association remains poorly understood. An assessment of the direction of the association can be obtained within a model that simultaneously considers both longitudinal paths, i.e. from physical activity to academic performance and from academic performance to physical activity. Moreover, very few studies have paid attention to the role of leisure-time physical activity, most have concentrated on physical activity organized in school, although leisure-time physical activity may better reflect voluntary behaviour than compulsory, school-based physical activity. Leisure-time physical activity also shows greater variation than school-based activities since the latter does not necessarily reflect the wishes and/or inherent abilities of the student.

Accordingly, our main aim was to investigate the direction and magnitude of the associations between leisure-time physical activity and academic performance at four time points across adolescence and young adulthood. In this way, we aimed to investigate whether leisure-time physical activity would be predictive for subsequent academic performance and/or educational attainment or vice versa, or both, and whether the possible associations would be sustained after the child leaves school and enters into young adulthood and early working life. Based on extensive previous evidence, we hypothesized that there would be a positive association between leisure-time physical activity and academic performance, i.e. that leisure-time physical activity would predict subsequent academic performance. We also aimed to examine whether environmental (e.g. family environment) and genetic factors shared by co-twins explain the association between leisure-time physical activity and academic performance. Furthermore, we aimed to investigate to what extent cognitive ability explains the association between leisure-time physical activity and educational attainment in young adulthood.

## Results

Descriptive statistics for leisure-time physical activity and academic performance are presented in Table 1. The most common grade point average for 12 and 14 year old participants was 8–9 (47.3%) with the maximum grade point average being 10. The majority of the participants (61.8%) were studying at upper secondary school at age of 17 years, and the most common educational attainment in young adulthood was tertiary education (53.3%). The most often reported frequency of leisure-time physical activity was 2–3 times a week in each study wave among participants (percentages ranged from 30.0% to 49.4%).

Academic performance variables were highly statistically significantly and positively correlated with each other across survey waves (Table 2); polychoric correlations ranged from 0.55 (p < 0.001) to 0.84 (p < 0.001). Similarly, leisure-time physical activity variables were statistically significantly positively correlated from one survey wave to the next, but the correlations were not as high as for academic performance variables (from 0.18 (p < 0.001) to 0.43 (p < 0.001)). Importantly, leisure-time physical activity and academic performance were also statistically significantly positively associated at the different survey points (range from r = 0.07 (p < 0.01) to r = 0.21 (p < 0.001)). The strongest associations were found between leisure-time physical activity behaviour at age of 17 years and academic performance at ages of 14 (r = 0.17, p < 0.001), 17 (r = 0.19, p < 0.001) and 24 (r = 0.21, p < 0.001) years as well as between leisure-time physical activity behaviour at age of 24 years and academic performance at age of 24 years (r = 0.15, p < 0.001) (Table 2).

**Figure 1:**
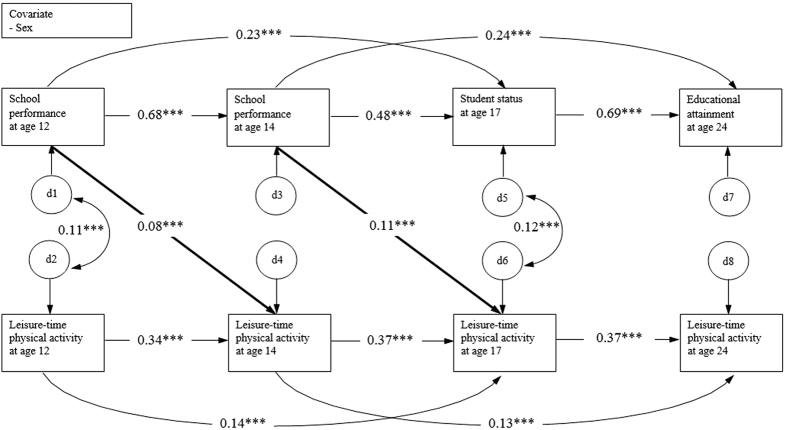
Cross-lagged path model for leisure-time physical activity and academic performance. Only participants’ sex was included in the model as a covariate and only statistically significant associations are presented in the figure. d = residual variance, ***p < 0.001.

First, we conducted the cross-lagged path model adjusting for the participants’ sex (Fig. 1). We observed that academic performance at each survey wave statistically significantly predicted subsequent academic achievements (standardized path coefficients from 0.48 (p < 0.001) to 0.69 (p < 0.001)). The same was seen between the repeated measurements of leisure-time physical activity over time. The coefficients were somewhat lower than those for academic performance, but remained statistically significant (standardized path coefficients from 0.34 (p < 0.001) to 0.37 (p < 0.001)). This auto-regressive part of the model indicates that the temporal stability of academic performance from the age of 12 years to the age of 24 years is higher than the temporal stability of leisure-time physical activity over the same time period.

The cross-lagged effects, which were crucial to our hypothesis indicated that higher academic performance at ages of 12 and 14 years predicted a statistically significantly higher frequency of leisure-time physical activity in the follow-up time-points, even if the previous level of leisure-time physical activity was taken into account in the model; standardized path coefficients were 0.08 (p < 0.001) at age 14 and 0.11 (p < 0.001) at age 17. This pattern was not found in young adulthood, and leisure-time physical activity did not predict subsequent academic performance at any time point. Residual correlations between leisure-time physical activity and academic performance at ages of 12 and 17 years were statistically significant (standardized path coefficients of 0.11 (p < 0.001) and 0.12 (p < 0.001)), respectively. The model had an excellent fit to the data (χ^2^ [7] = 18.23, *p* = 0.01, CFI = 0.999, RMSEA = 0.017).

**Figure 2:**
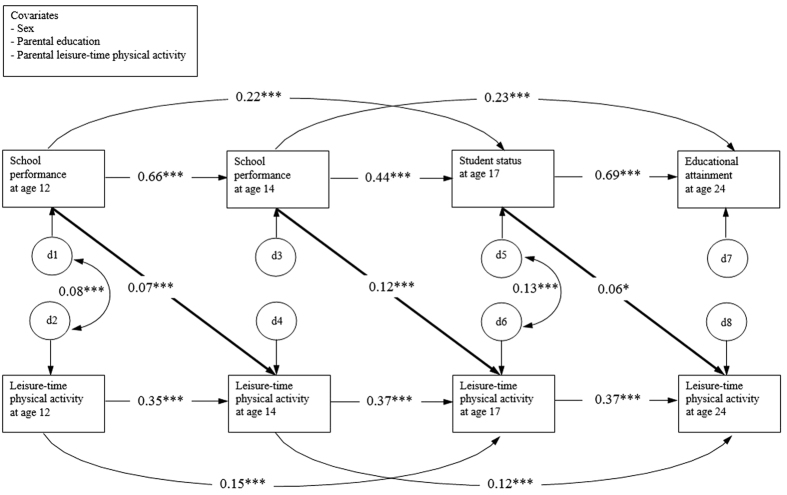
Cross-lagged path model for leisure-time physical activity and academic performance. The covariates of sex, parental education and parental leisure-time physical activity were included in the model. Only statistically significant associations are presented in the figure. d = residual variance, *p < 0.05, ***p < 0.001.

When the cross-lagged path model was adjusted for the participants’ sex, parental education and parental leisure-time physical activity, very few changes were seen compared to the analyses adjusted only for sex (Fig. 2). Previous academic performance again positively predicted the subsequent frequency of physical activity in leisure-time at age 14 (standardized path coefficient 0.07 (p < 0.001)) and at age 17 (standardized path coefficient 0.12 (p < 0.001)). One major change was that higher academic performance at age of 17 years predicted a statistically significantly higher frequency of leisure-time physical activity also at age of 24 years (standardized path coefficient was 0.06 (p < 0.05)). Again, leisure-time physical activity was not a predictor of academic performance at any single time point. Minor effects on the auto-regressive coefficients were observed. Residual correlations between leisure-time physical activity and academic performance at age of 12 and 17 years were statistically significant (standardized path coefficients of 0.08 (p < 0.001) and 0.13 (p < 0.001), respectively). The cross-lagged path model with covariates displayed also an excellent fit to the data (χ^2^ [7] = 17.12, *p* = 0.029 CFI = 0.999, RMSEA = 0.016).

Results of the bivariate cross-lagged path model for the within-pair differences in leisure-time physical activity and academic performance suggested that environmental and genetic factors shared by co-twins did not explain the association between leisure-time physical activity and academic performance: within-pair differences in academic performance positively predicted subsequent within-pair differences in leisure-time physical activity at age 14 (standardized path coefficients 0.06 (p < 0.001)) and at age 24 (standardized path coefficients 0.09 (p < 0.05)) being consistent with the individual-based results (Supplementary material II, Supplement Fig. 1).

Linear regression results for cross-sectional data on leisure-time physical activity, educational attainment and cognitive ability showed that a statistically significant and independent association between leisure-time physical activity and academic performance remained even after cognitive ability was included in the model (Supplementary material III).

## Discussion

We investigated the direction and magnitude of the associations between leisure-time physical activity and academic performance from adolescence to young adulthood in a cross-lagged path model. The results revealed a consistent pattern in which better academic performance in adolescence was modestly associated with an increased frequency of leisure-time physical activity in late adolescence and young adulthood, and that patterns was independent of the prior level of leisure-time physical activity as well as parental factors. The examination of the association from the reverse direction, i.e. whether leisure-time physical activity predicted academic performance, was also clear: we found no evidence for a link in any survey wave. However, it should be noted that there was relatively less variance in academic performance to be explained by leisure-time physical activity than the other way around, which may have affected the result.

Our results are consistent with recent studies and reviews confirming the positive association between physical activity and academic performance^4^,^5^,^9^,^10^. However, we were unable to demonstrate, and thus failed to confirm our working hypothesis, that physical activity would predict academic performance, as claimed in the review of Lees *et al*.^11^. Although that previous review of randomized controlled trials found a positive association, it was only a weak relationship. Our study design cannot prove causal relationships^18^, but because of its longitudinal design with several follow-up waves, our results are nevertheless informative about potential causal effects i.e. an appropriate temporal order is a necessary prerequisite for causality. Further, we demonstrated similar association within pairs of co-twins who share many childhood environmental factors and in the case of MZ twins, they are also genetically identical. This result suggests that the association between academic performance and physical activity is not explained by genetic and non-genetic factors shared by co-twins.

We also found that cognitive ability explained part of the association between educational attainment and leisure-time physical activity in young adulthood, but an independent association between academic performance and physical activity still remained although it was somewhat weaker. The reduced association was expected as cognitive ability is a major predictor of educational achievement and has been linked with physical activity levels^5^. The process of neuroselection^19^,^20^ may be one of the mechanisms that could explain why cognitive ability is associated with physical activity. The neuroselection hypothesis states that healthier behaviour choices are selected by the individuals with better cognitive ability. In other words, those who are cognitively more capable may have better learning and reasoning skills, and thus, they may be able to do better choices related to physical activity behaviour than those with lower cognitive ability^15^.

Very few previous studies have concentrated on leisure-time physical activity when studying associations between physical activity and academic performance. Our results with leisure-time physical activity, however, further support the positive association found between physical activity and academic performance in the reviews, which have concentrated on school-based physical activity^5^,^9^,^10^. Moreover, our study had one of the longest follow-ups in this field encompassing the transition from school to young adulthood and initiation of the working life. The results we found over the age range of secondary school students were consistent with the results found in recent studies^5^,^9^,^10^ as well as with confirming our own survey for follow-up time points from ages 12 to 17.

When comparing the results of different studies it is important to bear in mind that both physical activity and academic performance are complex to define and can be measured in many ways. For example, many of the recent studies revealing no association or negative results originate from studies, which measured physical activity behaviour objectively^12^,^13^,^14^. Methodological differences along with small sample sizes and the poor quality of many existing studies may also have resulted in the discrepant findings. It can be speculated that the duration of interventions may be another factor explaining the diverging results. Therefore, the results from different studies need to be compared with caution.

Varying results between studies could be due to national differences in the schooling systems. Finland offers an education that is publicly funded, and free to all children, which means that differences between schools are only marginal. PISA studies during the last decades have revealed that Finnish students have been very successful in their assessments^21^. PISA study focuses on students’ ability to use their knowledge and skills to meet real-life challenges. In addition, Finland is an active sporting nation adhering to the principle of “sport for all”^22^. Most Finnish young people have similar opportunities to exercise in their leisure-time. More than eight out of 10 Finns aged 15 and older engage in some kind of sporting activity^23^. Thus, the Finnish results are not explained by the heterogeneity of the educational system or exercise opportunities. Therefore, the conflicting results between previous publications and our current study may be explained by different educational and sport systems in different countries.

One of the main potential limitations of the present study is the use of self-reported questionnaire data to estimate: 1) leisure-time physical activity, 2) student status at age of 17 years and 3) educational attainment in young adulthood. It is well-known that self-reports may have shortcomings with respect to validity and the reliability of the measure^24^. Although the validity of the physical activity questionnaires used in Finnish twins has been demonstrated^25^,^26^,^27^,^28^, the possibility of errors cannot be avoided when using a non-objective instrument. In contrast to these subjective measurements, grade point averages in adolescence were reported by teachers when the twins were aged 12 and 14 years. One advantage of the teacher-based measurement is that a well-trained, professional has evaluated the twins’ school performance as objectively as possible, in the context of a very homogeneous school system with an overall national curriculum. In addition, the proficiency level of the teachers is rather similar since all have undergone Master’s level training.

This study was limited by the absence of an objective measure of leisure-time physical activity, and furthermore, criticism can be raised at the subjective assessment of physical activity. Limiting the subjective assessment of physical activity to only its frequency measure was not the most optimal way to measure physical activity behaviour. However, the frequency of physical activity was the only variable for which longitudinal data were available in the present study. It is possible that a lack of comprehensiveness in the assessment of leisure-time physical activity dimensions does somewhat restrict the picture of the total leisure-time physical activity behaviour. It is, however, unlikely that issues related to the measurement of physical activity would explain the association between physical activity and academic performance, instead, they would tend to make the association weaker.

Moreover, our findings are limited to people over 12 years of age. Thus, the direction of the association between leisure-time physical activity and academic performance among individuals under the age of 12 years remains still poorly known. It may be that the pattern of associations in early childhood is not consistent with the pattern we found among twins over 12 years of age. For example, the process of neuroselection has been suggested to occur more likely from adolescence onwards than in childhood^19^.

A key strength of the present study is the longitudinal study design with four waves, providing an opportunity to assess the mutual relationships between leisure-time physical activity and educational achievement. Longitudinal studies are ideal for studying factors over time, but most importantly, they enable testing the temporal directions of the association which is a prerequisite for the investigation of potential causal associations.

A further strength of this study is its large sample size, which ensures sufficient statistical power to detect statistically significant associations. Moreover, various selection biases are unlikely in our study due to the rather high participation rate in the survey and the inclusion of multiple domains in the questionnaire. We were able to take several covariates into account and therefore to control for potential familial confounding. In addition, we could adjust the results for unmeasured environmental and genetic factors by using the twin design, and also adjust the results for cognitive ability. Finally, our results are based on a population-based sample with relatively equal sex representation and high response rates, which contributes to the good generalizability of the study findings.

This study demonstrated that a better academic performance in adolescence modestly predicts an increased level of leisure-time physical activity in late adolescence and young adulthood. However, we found that leisure-time physical activity did not predict later academic performance at any time point in our survey. Thus, our findings do not clearly support the belief that physically active adolescents would perform better academically in the future, but rather children and adolescents who perform well in school tend to be more physically active later on.

For future studies, more information on the role of leisure-time physical activity instead of school based physical activity in the field is needed. Intervention studies, especially large randomized controlled trials, would also help to develop a full picture of the association between physical activity and academic performance. In public health perspective, the main implication of the present study is the need for better-targeted physical activity promotion actions for children and adolescents with lower levels of academic performance. Early detection and support may minimize the risk of lower levels of physical activity in later life. Such early-targeted interventions are also expected to be cost-effective, which is in the interest of policy makers.

## Methods

### Participants

The participants were drawn from the FinnTwin12 (FT12) study, which is a longitudinal study of health and behaviour in Finnish twins (birth cohorts 1983–1987) and their families^29^. The twins were identified from the Central Population Registry of Finland, and the relevant data for the study were collected through mailed questionnaires. The parents of twins assessed different aspects of their children’s behaviour and development as well as their own socioeconomic status and health behaviour. In addition, teachers assessed twins’ behaviour and academic performance at school.

To date, four waves of the FT12 study have been completed. In the first phase, the twins and their parents completed questionnaires when the twins were 11–12 years old. Subsequently, the twins were surveyed again at the mean ages of 14 (SD 0.08 years, range 13.7–14.9) and 17.6 (SD 0.26 years, range 17.2–19.5) years^30^,^31^. The last data collection wave of the FT12 study was conducted during the years 2006–2009 when the twins were young adults, at the mean age of 24.2 years (SD 1.64 years, range 20.5–27.5). The response rates have been high in each wave of data collection, ranging from 73% to 90%^29^,^31^.

Initially, 5,600 twins and their families were enrolled to the FT12 study. In the present study, we had data available on leisure-time physical activity and academic performance from 4,180 twin individuals (50% females) when they were 12 years old, 2,859 twin individuals (51% females) at age 14 years, 4,190 twin individuals (52% females) at age 17 years, and 3,171 twin individuals (61% females) they were 24 years old. Furthermore, we had data from 1,915, twin pairs (672 monozygotic (MZ) pairs, 634 dizygotic (DZ) same-sex pairs, 609 DZ opposite-sex pairs) at age 12, 1,174 twin pairs (427 MZ pairs, 384 DZ same-sex pairs, 363 DZ opposite-sex pairs) at age 14, 1,925 twin pairs (672 MZ pairs, 636 DZ same-sex pairs, 617 DZ opposite-sex pairs) at age 17, 1,261 twin pairs (489 MZ pairs, 392 DZ same-sex pairs, 380 DZ opposite-sex pairs) at age 24. In addition, we had cross-sectional data on general cognitive ability from 788 twin individuals at age 24 years.

### Assessment of leisure-time physical activity

Self-reported leisure-time physical activity behaviour was assessed for each study wave of the FT12 study. The assessment of twins’ activity level was based on a structured question on the frequency of leisure-time physical activity excluding physical education classes at school. The item was asked in exactly the same form in all survey waves “How often do you exercise or take part in sports during your leisure-time”? However, the response options were somewhat different. At the study wave of ages 12 and 14 years, there were five response options: (1) just about every day, (2) two to three times a week, (3) two to three times a month, (4) two to three times in six months, (5) not at all. When the twins aged, the seven response options at age of 17 years were: (1) just about every day, (2) four to five times a week, (3) two to three times a week, (4) about once a week, (5) one to two times a month, (6) less than once a month, (7) not at all. The options were the same in young adulthood as they were at age of 17 years, but one extra response option was included i.e. “several times every day”. Previous studies have shown good correlations between the leisure-time physical activity items used in the Finnish twin studies (including the question we used to assess leisure-time physical activity behaviour) and physical activity data obtained by interview (r = 0.56, p < 0.001)^25^ or by a detailed assessment of 12-month leisure-time physical activity history (r = 0.73, p < 0.001)^32^.

### Assessment of academic performance

School performance was assessed by teachers at school with a grade point average at ages of 12 and 14 years. Teachers answered the following question: “What was the twin’s grade point average at the end of the academic year? (in the Finnish school system, grade point averages range from the lowest value of 4 to the highest value of 10)”. The responses were categorized as following: (1) better than 9, (2) from better than 8 to 9, (3) from better than 7 to 8, (4) from better than 6 to 7, (5) six and under, (6) numeral grades not given. For the twins for whom numeral grades were not given at ages 12 (N = 1,275) or 14 (N = 12) years, their most likely grade point average category membership was imputed by using several other school performance variables reported by the teachers, such as spelling, reading aloud, and mathematics^33^. These estimations did not bias the associations with leisure-time physical activity (Supplementary material I).

At the age 17 years, the twins themselves reported their current student status, which was used as a measure of academic performance at that stage of their lives. The responses for this item in our survey were classified into three groups: (1) not studying currently, (2) in vocational school, (3) in academically oriented upper secondary school. The self-reported highest educational degree achieved at the age of 24 years was the measure of academic performance in young adulthood. For those twins who had not completed their education, we treated their ongoing studies as the final level of education. Educational attainment was classified into four groups: (1) compulsory education only, (2) vocational secondary education, (3) upper secondary education, (4) tertiary education (university or polytechnic college).

### Within-pair differences

Twin data allow us to determine whether environmental or genetic factors shared by co-twins explain the association between leisure-time physical activity and academic performance. This was achieved by creating new variables for within-pair differences in leisure-time physical activity and academic performance at all four survey waves (Supplementary material II). The variables were constructed by subtracting the observations from one co-twin from the observations of the other co-twin to obtain a single value representing the difference between co-twins in their physical activity and academic performance traits. Higher values represent a greater difference between co-twins whereas zero means that there is no difference between co-twins.

### Covariates

The cross-lagged path analyses were adjusted for sex. In additional analyses to diminish potential confounding by familial factors, we adjusted the results for parents’ educational level and parents’ leisure-time physical activity behaviour reported separately by the mother and father. Parental education was measured at baseline when the twins were 12 years. We used the educational level of the more highly educated parent classified into four categories similar to those used in assessing the twins. Parents’ self-reported physical activity was assessed as the frequency of leisure-time physical activity similar to the physical activity assessment in twins. The structured question used for parents was: “How many times a month do you exercise”? The options for the response were (1) less than once a month, (2) 1–2 times a month, (3) 3–5 times a month, (4) 6–10 times a month, (5) 11–20 times a month, (6) over 20 times a month.

Because cognitive ability may explain a part of the association between physical activity and academic performance, we wanted to determine whether this would apply to our data (Supplementary material III). We had data on the participants’ general cognitive ability, also known as general intelligence from the fourth study wave at age 24 years based on the Wechsler Adult Intelligence Scale III test^34^. The scale provided scores for verbal and performance intelligence, which were used to derive our measure of general cognitive ability.

### Statistical methods

We started the analyses by calculating correlations between leisure-time physical activity and academic performance by using Mplus statistical software package, version 5.2^35^. We used polychoric correlations to estimate the associations between two theorized normally distributed continuous latent variables from two observed ordinal variables. Polychoric correlations were also used for the variables that represented within-pair differences in leisure-time physical activity and academic performance (Supplementary material II, Supplement Table 1).

**Figure 3:**
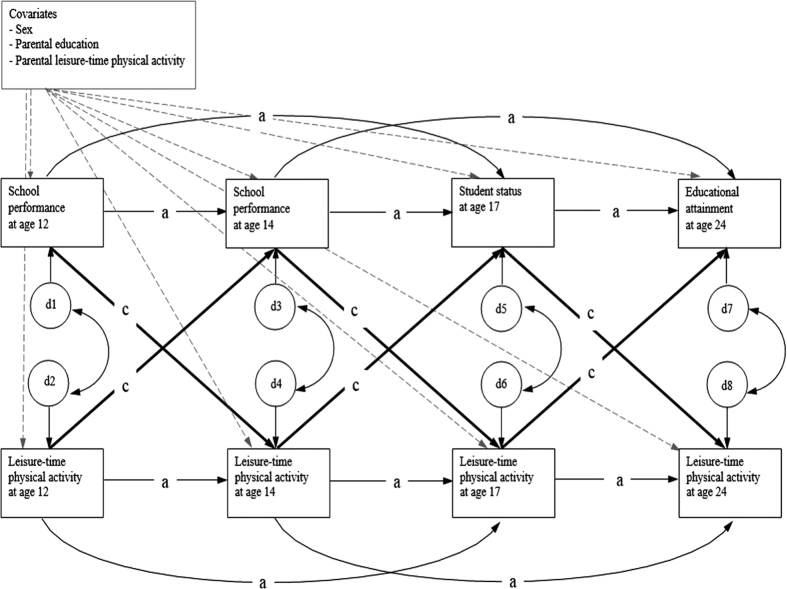
Cross-lagged path model for leisure-time physical activity and academic performance. a = auto-regressive path, c = cross-lagged path, d = residual variance.

Twins were analyzed as individuals in our main analyses, and because it is likely that the observations between co-twins of a twin pair will be correlated, twin clustering was controlled for by adjusting the standard errors for the clustering of observations obtained in twin pairs using the type = complex specification and the cluster option implemented in Mplus^35^. This option uses the pseudomaximum likelihood method in which the asymptomatic covariance matrix of the estimates is obtained by a robust sandwich estimator^36^. A bivariate cross-lagged path model was fitted for leisure-time physical activity and academic performance across the four study waves. The cross-lagged path model was used to estimate the structural relations of repeatedly measured variables. The auto-regressive part of the model indicates the temporal stability of the variables from one occasion to the next (also additional auto-regressive paths on variables at waves 3 and 4 from the equivalent measures two waves earlier were used to improve the model fit) while cross-lagged paths are used to assess reciprocal relationships between the variables at consecutive time points, i.e. leisure-time physical activity and academic performance during the follow-up. The cross-lagged path model can incorporate covariates and, thus, we fitted the model both with and without covariates. Residual correlations at each study wave were also modeled. Residual correlations between leisure-time physical activity and academic performance denote cross-sectional correlations between these two traits not explained by the cross-lagged associations. The full cross-lagged path model is shown in Fig. 3.

We used weighted least squares estimation for ordinal variables in Mplus^35^. This estimator allows missing data as a function of the observed covariates. Data are assumed to be missing at random with respect to the outcome variables, but missingness was allowed to be dependent on the covariates. Without any covariates, the model is similar to a pairwise analysis in which all available data are used to estimate pairwise associations. The weighted least squares estimation has been shown to be consistent and more efficient than the estimators based on a listwise deletion, especially when the amount of missing data is not substantial^37^.

In addition to individual-based analyses, a bivariate cross-lagged path model was also fitted for within- pair differences in leisure-time physical activity and academic performance across the four study waves (Supplement Fig. 1). Twin clustering was not controlled, nor were covariates used since the creation of the within-pair variables takes these adjustments into account.

Several statistical tests were used to determine how well the model fits our data. Chi-square tests of model fit were computed to indicate the difference between observed and expected covariance matrices. The non-independence of observations due to cluster sampling was taken into account. Chi-square tests values closer to zero indicate a better fit, i.e. a smaller difference between expected and observed covariance matrices. Another indicator of model fit was the comparative fit index (CFI), which has a scale of 0 to 1; values greater than 0.9 are generally indicative of an acceptable model fit^38^. Finally, the root mean square error of approximation (RMSEA) ranging also from 0 to 1 was used; values below 0.5 are taken to indicate that the model is plausible.

We used Stata 13.0 (StataCorp, College Station, Texas) to examine to what extent cognitive ability would explain the association between leisure-time physical activity and educational attainment. A linear regression analysis was used to study the associations between leisure-time physical activity, educational attainment and cognitive ability. Stata’s cluster option was applied to take account of possible within-pair correlations.

### Ethics of the study

The ethics committee of the Department of Public Health of the University of Helsinki (Helsinki, Finland), the ethics committee of the Helsinki University Central Hospital District (Helsinki, Finland) and the Institutional Review Board of Indiana University (Bloomington, Indiana, USA) approved the FT12 study protocol. All study methods were carried out in accordance with the approved guidelines. The parents of the participating twins or twins themselves as young adults provided written informed consent for participation in the study.

### Data Availability

Due to the consent given by study participants and the ease of identifying twins, data cannot be made publicly available. Data are available through the Institute for Molecular Medicine Finland (FIMM) Data Access Committee (DAC) for authorized researchers who have IRB/ethics approval and an institutionally approved study plan. For more details, please contact the FIMM DAC (fimm-dac@helsinki.fi).

## Additional Information

**How to cite this article**: Aaltonen, S. *et al*. Leisure-Time Physical Activity and Academic Performance: Cross-Lagged Associations from Adolescence to Young Adulthood. *Sci. Rep.* **6**, 39215; doi: 10.1038/srep39215 (2016).

**Publisher's note:** Springer Nature remains neutral with regard to jurisdictional claims in published maps and institutional affiliations.

## Supplementary Material

Supplementary Information

## Figures and Tables

**Table 1:** Descriptive statistics for the frequency of leisure-time physical activity and academic performance.

Characteristics	Number of participants (percentage)	Characteristics	Number of participants (percentage)
Leisure-time physical activity at age 12	N = 5,105	School performance at age 14	N = 2,923
just about every day	670 (13.1%)	grade point average better than 9	299 (10.2%)
two to three times a week	2,524 (49.4%)	grade point average 8–9	1,273 (43.6%)
two to three times a month	366 (7.2%)	grade point average 7–8	979 (33.5%)
two to three times in six months	222 (4.4%)	grade point average 6–7	346 (11.8%)
not at all	1,323 (25.9%)	under 6	26 (0.9%)
Leisure-time physical activity at age 14	N = 4,715	Student status at age 17	N = 4,207
just about every day	949 (20.1%)	not studying currently	182 (4.3%)
four to five times a week	814 (17.3%)	in vocational school	1,426 (33.9%)
two to three times a week	1,418 (30.1%)	in upper secondary school	2,599 (61.8%)
about once a week	818 (17.4%)	Educational attainment at age 24	N = 3,218
one to two times a month	364 (7.7%)	compulsory education	128 (4.0%)
less than once a month	207 (4.4%)	vocational secondary education	917 (28.5%)
not at all	145 (3.1%)	upper secondary education	457 (14.2%)
Leisure-time physical activity at age 17	N = 4,218	tertiary education	1,716 (53.3%)
just about every day	800 (19.0%)	Sex	N = 5,398
four to five times a week	717 (17.0%)	male	2,724 (50.5%)
two to three times a week	1,266 (30.0%)	female	2,674 (49.5%)
about once a week	686 (16.3%)	Parental education	N = 4,927
one to two times a month	326 (7.7%)	compulsory education	1,479 (30.0%)
less than once a month	196 (4.7%)	vocational secondary education	1,417 (28.8%)
not at all	227 (5.4%)	academic secondary education	1,329 (27.0%)
Leisure-time physical activity at age 24	N = 3,377	tertiary education	702 (14.3%)
several times every day	83 (2.5%)	Maternal leisure-time physical activity	N = 5,100
just about every day	390 (11.6%)	over 20 times a month	875 (17.2%)
four to five times a week	556 (16.5%)	11–20 times a month	1,046 (20.5%)
two to three times a week	1,114 (33.0%)	6–10 times a month	1,310 (25.7%)
about once a week	641 (19.0%)	3–5 times a month	825 (16.2%)
one to two times a month	314 (9.3%)	1–2 times a month	550 (10.8%)
less than once a month	208 (6.2%)	less than once a month	494 (6.7%)
not at all	71 (2.1%)	Paternal leisure-time physical activity	N = 4,583
School performance at age 12	N = 4,372	over 20 times a month	557 (12.2%)
grade point average better than 9	154 (3.5%)	11–20 times a month	768 (16.8%)
grade point average 8–9	2,067 (47.3%)	6–10 times a month	1,133 (24.7%)
grade point average 7–8	1,816 (41.5%)	3–5 times a month	834 (18.2%)
grade point average 6–7	323 (7.4%)	1–2 times a month	624 13.6%)
under 6	12 (0.3%)	less than once a month	667 (14.6%)

**Table 2:** Polychoric correlations for leisure-time physical activity and academic performance from age 12 to age 24 years (four time points).

	School performance at age 12	School performance at age 14	Student status at age 17	Educational attainment at age 24	Leisure-time physical activity at age 12	Leisure-time physical activity at age 14	Leisure-time physical activity at age 17	Leisure-time physical activity at age 24
School performance at age 12	1 (N = 4,372)							
School performance at age 14	0.70*** (N = 2,553)	1 (N = 2,923)						
Student status at age 17	0.56*** (N = 3,512)	0.63*** (N = 2,576)	1 (N = 4,207)					
Educational attainment at age 24	0.55*** (N = 2,657)	0.67*** (N = 1,968)	0.84*** (N = 2,811)	1 (N = 3,218)				
Leisure-time physical activity at age 12	0.10*** (N = 4,180)	0.07** (N = 2,835)	0.10*** (N = 4,151)	0.12*** (N = 3,089)	1 (N = 5,105)			
Leisure-time physical activity at age 14	0.12*** (N = 3,892)	0.13*** (N = 2,859)	0.12*** (N = 4,148)	0.08*** (N = 3,005)	0.34*** (N = 4,555)	1 (N = 4,715)		
Leisure-time physical activity at age 17	0.12*** (N = 3,520)	0.17*** (N = 2,580)	0.19*** (N = 4,190)	0.21*** (N = 2,819)	0.26*** (N = 4,161)	0.43*** (N = 4,159)	1 (N = 4,218)	
Leisure-time physical activity at age 24	0.08*** (N = 2,766)	0.10*** (N = 2,023)	0.14*** (N = 2,932)	0.15*** (N = 3,171)	0.18*** (N = 3,224)	0.29*** (N = 3,128)	0.43*** (N = 2,941)	1 (N = 3,377)

The number of participants is given in brackets. ^**^p < 0.01, ^***^p < 0.001.
