# Supplementary material for: Leisure-Time Physical Activity and Academic Performance: Cross-Lagged Associations from Adolescence to Young Adulthood
Source: Sci Rep. 2016 Dec 15;6:39215. doi: 10.1038/srep39215 (PMC5156951; doi:10.1038/srep39215)
Supplement: Supplementary Information [file srep39215-s1.pdf]

SUPPLEMENTARY INFORMATION

Leisure-Time Physical Activity and Academic Performance: Cross-Lagged Associations from Adolescence to Young Adulthood

Sari Aaltonen*, Antti Latvala, Richard J. Rose, Urho M. Kujala, Jaakko Kaprio, Karri Silventoinen

SUPPLEMENTARY MATERIAL 1

Imputation of grade point average values

A standard grading system is used in Finland, but not all schools give numerical grades during the first comprehensive school years. In the present sample, teachers of 1,249 twin participants at age 12 reported that numerical grades were not given. For these participants we imputed their most likely grade point average category based on several school performance measures reported by the teachers, as was done in an earlier study[1]. We used a total of seven academic and behavioral measures (i.e., spelling, writing essays, reading aloud, comprehension, mathematics, diligence, and attentiveness), reported by the teachers, as predictors of the ordinal grade point average variable in a multinomial logistic regression model. Correlations between these measures and grade point average were between 0.59 and 0.69. Based on the pseudo-R2 statistic, the measures collectively explained 44% of the variation in the grade point average variable in the multinomial logistic model.

We used the post-estimation command with the outcome option in Stata 12 (StataCorp, College Station, Texas)[2] to estimate the probability of each category and then selected the most likely category for each individual with a missing grade point average value. Of the 1,249 twin participants, 616 participants (49.3%) were imputed as having a grade point average from better than 8 to 9, 568 participants (45.5%) as having a grade point average from better than 7 to 8, and 65 participants (5.2%) as having a grade point average from better than 6 to 7. These proportions were in line with the grade point average distribution in the full sample where the corresponding proportions were 8.3%, 40.0%, and 46.5%, respectively. The predicted probabilities of belonging to these categories ranged from 0.83 to 0.93.

To check for any potential bias introduced by the imputation, we compared the correlations of the original (non-imputed) and the final (imputed and non-imputed) grade point average variables with leisure-time physical activity measures at age 12. The correlations were similar (0.03 and 0.04, respectively), suggesting that no bias was present.

References

1. Latvala, A., et al. Drinking, smoking, and educational achievement: cross-lagged associations from adolescence to adulthood. *Drug Alcohol Depend.* **137**, 106–113 (2014).

2. StataCorp. Stata Statistical Software: Release 12. (StataCorp LP, 2011).

SUPPLEMENTARY MATERIAL 2

A cross-lagged path model for within-pair differences in leisure-time physical activity and academic performance

The aim of the supplementary analysis was to examine whether environmental and genetic factors shared by co-twins explain the association between leisure-time physical activity and academic performance. We created variables for within-pair differences in leisure-time physical activity and academic performance from the age of 12 to 24 years (N=2,682). The variables were constructed by subtracting the observations from one co-twin from the observations of the other co-twin. This was done to obtain a single value representing the difference between co-twins in their physical activity and academic performance traits. Higher values represent a greater difference between co-twins and zero means that that there is no difference between co-twins. The variable construction was done for all leisure-time physical activity and academic performance variables at all four study waves in order to take environmental and genetic factors shared by co-twins into account at every survey wave.

The analyses were conducted using Mplus statistical software package, version 5.2. We started the analyses by calculating polychoric correlations for the within-pair differences in leisure-time physical activity and academic performance (Supplement table 1). The correlations varied greatly across age: range was from no correlation to highly statistically significant correlation between within-pair differences in leisure-time physical activity and academic performance. The strongest associations were found between within-pair differences in leisure-time physical activity at age of 17 years and academic performance at age of 24 years (r=0.11, p<0.001), as well as between within-pair differences in leisure-time physical activity at age of 24 years and academic performance at age of 17 (r=0.11, p<0.01) and 24 (r=0.10, p<0.01) years.

A bivariate cross-lagged path model was fitted for the within-pair differences in leisure-time physical activity and academic performance across the four study waves (Supplement figure 1). The auto-regressive part of the model indicated that the within-pair differences in both leisure-time physical activity and academic performance statistically significantly predicted trait values from one study wave to the next (standardized path coefficients for leisure-time physical activity from 0.15 ($p<0.001$) to 0.24 ($p<0.001$), and for academic performance from 0.31 ($p<0.001$) to 0.57 ($p<0.001$)).

The cross-lagged effects of the model indicated that previous within-pair differences in academic performance positively predicted the subsequent within-pair difference in leisure-time physical activity at age 14 (standardized path coefficients 0.06 ($p<0.001$)) and at age 24 (standardized path coefficients 0.09 ($p<0.05$)) years being consistent with individual-based results, and indicating that environmental and genetic factors shared by co-twins do not have a crucial influence on the association between leisure-time physical activity and academic performance in our data. Previous within-pair differences in academic performance did not predict subsequent within-pair differences in leisure-time physical activity at age of 17 years.

Previous within-pair difference in leisure-time physical activity did not predict within-pair difference in later academic performance at age of 14 or 17. Within-pair difference in leisure-time physical activity at age of 17 years positively predicted within-pair difference in the final level of education in young adulthood (standardized path coefficients 0.06 ($p<0.05$)). No residual correlations between variables were found. To test how well the model fitted the data, the chi-square tests of model fit were computed, as well as the comparative fit index (CFI) and the root mean square error of approximation (RMSEA): the model had a very good fit to the data (χ^2 [8]=8.43, p=0.39, CFI=1.0, RMSEA=0.004).

Supplement table 1 Polychoric correlations for within-pair differences in leisure-time physical activity and academic performance from the age of 12 to 24 years (four time points). Sample sizes are given in brackets.

	School performance at age 12	School performance at age 14	Student status at age 17	Educational attainment at age 24	Leisure-time physical activity at age 12	Leisure-time physical activity at age 14	Leisure-time physical activity at age 17	Leisure-time physical activity at age 24
School performance at age 12	1 (N=2,008)							
School performance at age 14	.49*** (N= 1,053)	1 (N=1,212)						
Student status at age 17	.35*** (N=1,592)	.40*** (N=1,059)	1 (N=1,942)					
Educational attainment at age 24	.31*** (N=1,081)	.48*** (N=734)	.67*** (N=1,147)	1 (N=1,290)				
Leisure-time physical activity at age 12	-.002 (N=1,915)	-.002 (N=1,166)	-.03 (N=1,898)	.04 (N=1,240)	1 (N=2,379)			
Leisure-time physical activity at age 14	.07* (N=1,765)	-.01 (N=1,173)	.06** (N=1,900)	.04 (N=1,205)	.15*** (N=2,084)	1 (N=2,168)		
Leisure-time physical activity at age 17	.02 (N=1,592)	.06 (N=1,059)	.07** (N=1,925)	.11*** (N=1,147)	.11*** (N=1,899)	.25*** (N=1,902)	1 (N=1,944)	
Leisure-time physical activity at age 24	.04 (N=1,108)	.08* (N=750)	.11** (N=1,183)	.10** (N=1,261)	.05 (N=1,283)	.15*** (N=1,242)	.27*** (N=1,183)	1 (N=1,333)

*=p<0.05, **=p<0.01, ***=p<0.001

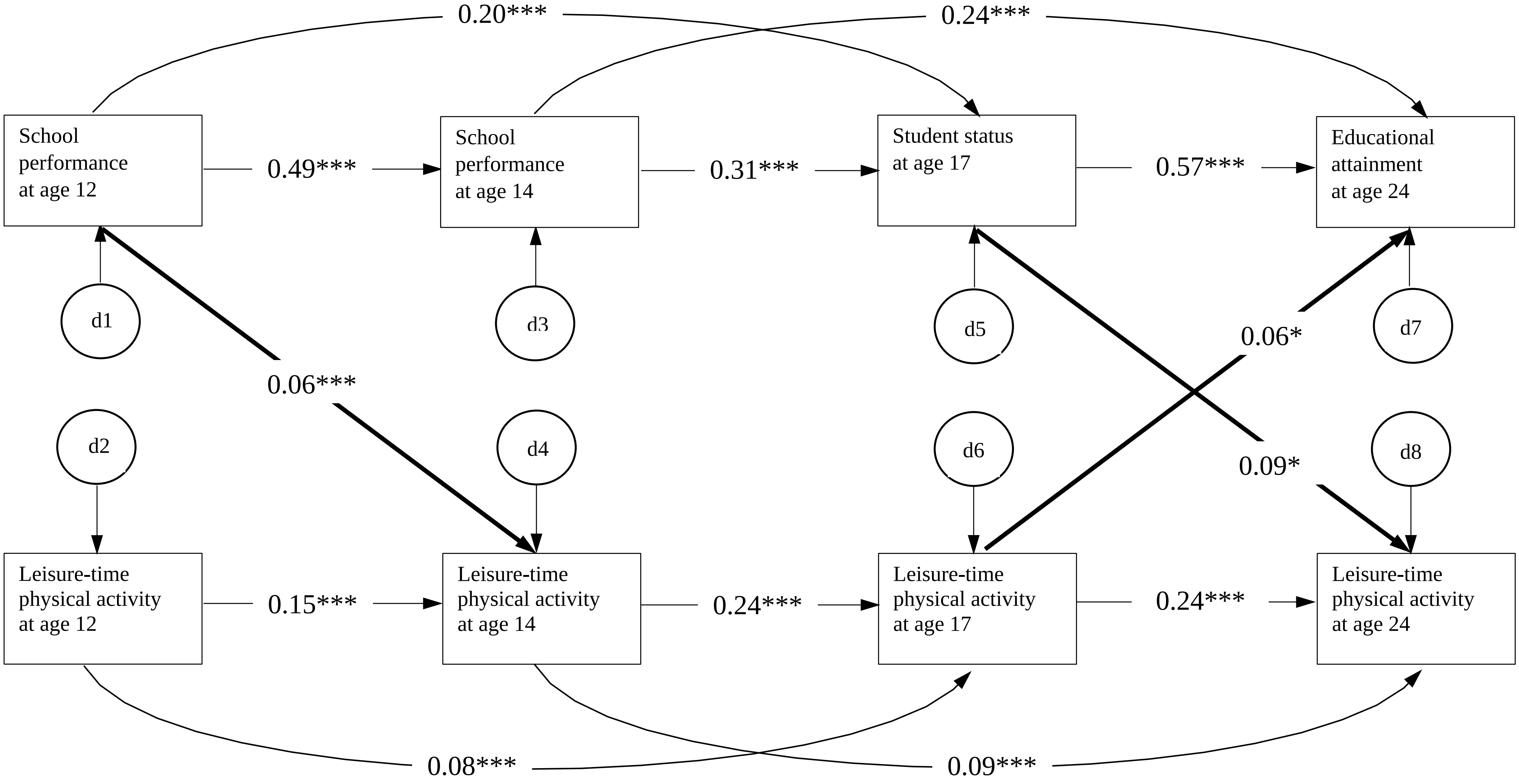

Supplement figure 1 Cross-lagged path model for within-pair leisure-time physical activity and academic performance. Only statistically significant associations are presented in the present figure. d = residual variance, *=p<0.05, **=p<0.01, ***=p<0.001.

SUPPLEMENTARY MATERIAL 3

Cross-sectional associations between leisure-time physical activity, educational attainment and cognitive ability in young adulthood

The aim of the supplementary analysis was to examine to what extent cognitive ability may explain the association between leisure-time physical activity and educational attainment in our data. We had cross-sectional data on leisure-time physical activity, educational attainment and cognitive ability from 788 twins taking part in the FT12 study. The data was collected at the study wave four when twins were young adults (mean age of 24.2 years). Wechsler Adult Intelligence Scale III was used to measure cognitive ability. The scale provided scores for verbal and performance intelligence. Based on these two scores we estimated general cognitive ability.

Statistical analyses were conducted using Stata 13.0 (StataCorp, College Station, Texas) and linear regression model was used to predict the association between leisure-time physical activity, educational attainment and cognitive ability. Stata's cluster option was used to take account for possible within-pair correlations. First, analyses without cognitive ability were conducted and educational attainment statistically significantly predicted leisure-time physical activity (β=0.21, SE=0.02, $p<0.001$). When cognitive ability was added to the model, all regression coefficients were positive indicating that not only educational attainment (β=0.16, SE=0.03, $p<0.001$), but cognitive ability (β=0.09, SE=0.002, $p=0.03$) was also positively associated with leisure-time physical activity level in young adulthood after taking into account participants' sex. However, the independent association between leisure-time physical activity and educational attainment remained even after cognitive ability was added to the model.

Participants' education, sex and cognitive ability accounted for 5.5% of the explained variability in leisure-time physical activity.

Reference

1. Wechsler, D. The Wechsler Adult Intelligence Scale (WAIS-III). Administration and Scoring Manual. 3rd ed. (The Psychological Corporation, 1997).
